# Electrochemical Monitoring in Anticoagulation Therapy

**DOI:** 10.3390/molecules29071453

**Published:** 2024-03-24

**Authors:** Ashwin K. V. Mruthunjaya, Angel A. J. Torriero

**Affiliations:** School of Life and Environmental Sciences, Deakin University, Burwood 3125, Australia

**Keywords:** electrochemical sensors, coagulation, anticoagulants, point of care, thrombin, factor Xa

## Abstract

The process of blood coagulation, wherein circulating blood transforms into a clot in response to an internal or external injury, is a critical physiological mechanism. Monitoring this coagulation process is vital to ensure that blood clotting neither occurs too rapidly nor too slowly. Anticoagulants, a category of medications designed to prevent and treat blood clots, require meticulous monitoring to optimise dosage, enhance clinical outcomes, and minimise adverse effects. This review article delves into the various stages of blood coagulation, explores commonly used anticoagulants and their targets within the coagulation enzyme system, and emphasises the electrochemical methods employed in anticoagulant testing. Electrochemical sensors for anticoagulant monitoring are categorised into two types. The first type focuses on assays measuring thrombin activity via electrochemical techniques. The second type involves modified electrode surfaces that either directly measure the redox behaviours of anticoagulants or monitor the responses of standard redox probes in the presence of these drugs. This review comprehensively lists different electrode compositions and their detection and quantification limits. Additionally, it discusses the potential of employing a universal calibration plot to replace individual drug-specific calibrations. The presented insights are anticipated to significantly contribute to the sensor community’s efforts in this field.

## 1. Introduction

The blood, a biofluid circulating within our bodies, serves various purposes, such as carrying oxygen and carbon dioxide and supplying essential nutrients to cells and tissues. Additionally, this circulating blood provides valuable insights into coagulation parameters, hypercoagulability, and changes in fibrinolysis. Blood coagulation is a process in which the circulating blood becomes a solid clot to arrest bleeding. It is a crucial process initiated in response to an injury, whether internal or external. Nonetheless, a disruption to this normal coagulation process, such as hypercoagulation, can cause excessive blood clot formation, which leads to blood vessel blockage, causing stroke [1,2]. The coagulation status can severely alter and induce severe complications in patients who have cancer, infectious diseases, trauma, diabetes, and retinal vein occlusion [3,4,5,6,7,8,9,10,11,12]. Furthermore, patients must take defined doses of anticoagulants to regulate blood clots in some pathological conditions. Therefore, it is critical to monitor blood coagulation to ensure blood is not clotting too rapidly or slowly.

Given the clinical importance of anticoagulant sensing, this review explores the research progress in this area. The working principle of various detection and quantification techniques for blood coagulation has been explained in the literature [13,14,15]. However, this review focuses on recent advances in coagulation monitoring systems exclusively based on electrochemical methods. In this review, we discussed (*i*) different steps in the blood clot process, such as platelet activation, the initiation of the coagulation cascade to form blood clots and the breakdown of blood clots, (*ii*) an introduction to traditional anticoagulants and the new generation of oral anticoagulants, (*iii*) a clot-based laboratory analysis used for coagulation monitoring, (*iv*) anticoagulant assays purely based on electrochemical detection methods which use thrombin activity or direct thrombin measurements, and (*v*) electrochemical sensors with direct measurements of anticoagulants depending on the redox behaviour of the drug or redox probes utilising electrode modification without the help of coagulant factors, such as thrombin or factor Xa.

## 2. Overview of Haemostasis

Haemostasis is a critical physiological function responsible for preserving blood flow under normal conditions and stopping blood loss after vascular damage [16]. This process is dependent on both cellular and plasma components. Typical haemostasis involves blocking the damaged blood vessel through platelet adhesion at the site of the injury, forming a fibrin clot to stabilise the blood loss, and finally, clot dissolution [17]. Moreover, all of these processes occur within a flow environment and are managed and governed by factors released from the adjacent endothelium. Therefore, it is essential to understand the different steps involved in haemostasis to design an assay that measures blood coagulation status.

### 2.1. Platelets

Platelets are essential to circulating blood and play an active role in haemostasis. Platelets are 2–4 µm in diameter and produced by a process called thrombopoiesis in the bone from megakaryocytes. Once formed in human blood, platelets remain circulating for approximately 7–10 days [18]. In an external stimulus, such as vascular injury to the blood vessels, platelets block the damaged blood vessel to prevent blood loss. This process is achieved by significant changes to the shape of the platelets (platelet activation), attachment to the subendothelial wall and other platelets, and the release of intracellular granules [19]. The initial phase of haemostasis involves the aggregation of platelets at the site of vascular damage under high blood flow conditions [20]. To accomplish this, platelets cling to collagen and plasma von Willebrand factor (vWf) due to the damaged endothelial cell wall. The interaction of the vWf and glycoproteins (GPIb-IX-V) occurs under high shear conditions and is believed to be a predominant receptor–ligand interaction that initiates platelet adhesion [21]. At the same time, collagen attachment occurs via GPVI and the α2β1 integrin complex, further delivering activation signals to platelets. Stable platelet adhesion is achieved through α5β1 to fibronectin or collagen binding at the vessel wall [20]. After the substantial accumulation of platelets, the delivery of adenosine diphosphate and thromboxane A2 attracts more platelets to the site of vascular damage. Such a phenomenon promotes platelet aggregation, causing a secondary accumulation [18]. Platelets also cause a sequence of enzymatic reactions known as the coagulation cascade [22]. When platelet activation occurs, the surface membrane becomes charged, and the subsequent exposure of inner leaflets further helps negatively charged phospholipids in the membrane create a conducive platform on which the coagulation cascade can occur when calcium ions are present [23].

### 2.2. Coagulation Cascade

**Figure 1:**
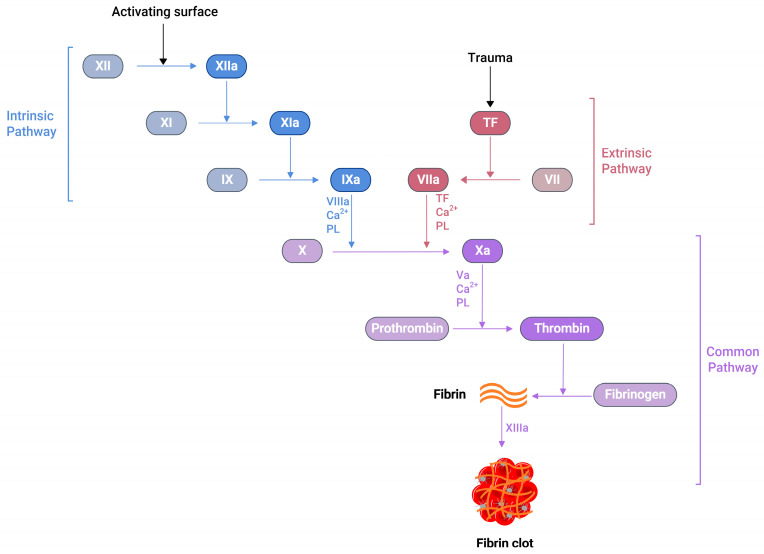
A schematic representation of the blood coagulation cascade. The plasma clotting reactions consist of two mechanisms: the intrinsic and extrinsic pathways. The extrinsic pathway is initiated by vascular injury and subsequent tissue factor (TF) exposure in the blood plasma. The formation of thrombin can activate the intrinsic pathway, which further activates FXI. Both pathways converge at the FXa formation, which produces a burst of thrombin at the end, converting fibrinogen to fibrin with the simultaneous activation of the platelets, resulting fibrin clot, which arrests blood loss.

Several soluble proteins in the blood plasma work together in a cascade of enzymatic reactions (Figure 1), culminating in the production of fibrin polymers and stabilising the blood clot [24]. When an injury occurs at the blood vessel wall, tissue factor (TF) becomes exposed alongside collagen in the subendothelial surfaces. Then, the exposed TF comes into contact with factor VIIa (FVIIa) in the plasma to produce the TF:FVIIa composite, also called extrinsic tenase [25]. This composite is the initial point in the coagulation cascade, referred to as an extrinsic or tissue factor pathway. This pathway ultimately transforms factor X (FX) to factor Xa (FXa) in vivo [25].

Similarly, another type of pathway exists in the coagulation cascade, known as the intrinsic or contact pathway (Figure 1). When damaged cell surfaces are in contact with high-molecular-weight plasma proteins, such as kininogen and kallikrein, the activation of factor XII (FXII) to factor XIIa (FXIIa) takes place, followed by further downstream conversions of factor XI (FXI) to factor XIa (FXIa), and factor IX (FIX) to factor IXa (FIXa) [24]. The activated FIXa forms a complex with factor VIIIa (FVIIIa) to produce intrinsic tenase complex FVIIIa:FIXa, which activates FX to FXa [25]. Independent of the extrinsic or intrinsic pathway, once FXa is released in the coagulation cascade, it forms a 1:1 ratio complex, prothrombinase, with factor Va (FVa), calcium ions, and phospholipid. Prothrombinase converts prothrombin to thrombin, resulting in the thrombin-mediated cleavage of fibrinogen to fibrin. Prothrombinase production and the subsequent thrombin and fibrin creation steps are described as the common pathways in the coagulation cascade (Figure 1) [25]. The final product of this common pathway (i.e., fibrin) plays a crucial role in creating a polymer fibrin gel. Then, factor XIIIa (FXIIIa), activated by thrombin, covalently crosslinks loosely bound fibrin gel, forming insoluble fibrin clots with platelets [25,26].

### 2.3. Thrombosis

Thrombosis is a phenomenon in which blood clots occur inside the blood vessel walls and is caused by the action of the coagulation cascade in response to pathological changes [24]. This excessive build-up of blood clots inside the lumen of a blood vessel can trigger acute complications, like deep vein thrombosis, pulmonary embolism, and myocardial infarctions, leading to potentially life-threatening conditions [27,28]. The process of platelet accumulation and thrombin generation through the coagulation cascade are functions of a healthy person. However, excessive unwanted clots can cause adverse effects. As a result, a down-regulating mechanism controls disproportionate platelet aggregation and thrombin generation, which is in balance with the normal haemostasis process. Naturally occurring anticoagulants, such as antithrombin III, protein C, and heparin cofactor II, are released when coagulation factors are activated [29,30]. Similarly, prostaglandin-I2 inhibits platelet activation to balance the prothrombic environment in healthy blood vessels [31].

Furthermore, once fibrin clots form at the site of the vessel injury, a counteracting fibrinolytic mechanism also exists to dissolve fibrin clots. When urokinase or a tissue-type plasminogen activator interacts with plasminogen, it initiates the conversion of plasminogen to plasmin. This plasmin subsequently dissolves fibrin clots and produces fibrin degradation products known as D-dimer [29,32].

## 3. Drugs Used in Anticoagulation Therapy

**Figure 2:**
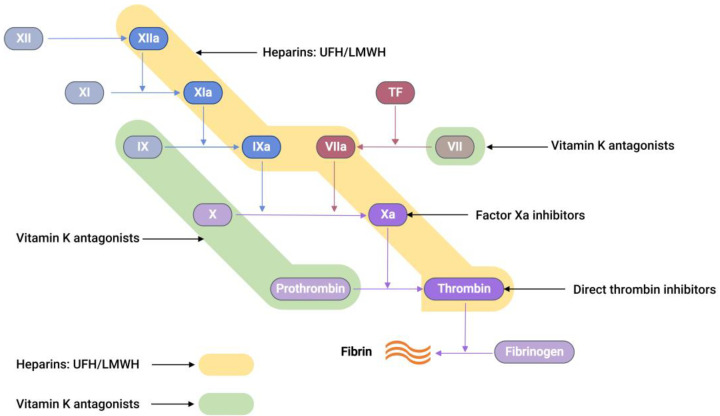
A schematic representation showing the sites of action of different anticoagulants. Unfractionated heparin (UFH) and low-molecular-weight heparin (LMWH) both bind to antithrombin-III (AT-III), resulting in a complex that is capable of inhibiting thrombin, factor Xa, TF-VIIa, factor IXa, factor XIa, and factor XIIa (highlighted in yellow) [35]. Vitamin K antagonists act as anticoagulants by inhibiting the synthesis of the active versions of four vitamin K-dependent procoagulant factors, including VII, IX, X, and prothrombin (highlighted in green) [36].

Commercially available anticoagulants work by either directly or indirectly modulating the actions of various proteins participating in the coagulation cascade system, thereby controlling blood coagulation (Figure 2).

Unfractionated heparin, UFH, an anticoagulant drug with an average molecular weight of 15 kDa, is generally extracted and purified from pig intestines [33]. UFH works on antithrombin III (AT-III), a naturally occurring glycoprotein that inhibits several clotting factors such as thrombin, factor Xa, TF-VIIa, factor IXa, and factor XIa to be active. A conformational change at the reactive arginine centre of AT-III induced by heparin interaction converts AT-III from a slow-acting inhibitor to a fast-acting inhibitor [34]. It was observed that the glucosamine unit and the pentasaccharide sequence are responsible for AT-III binding and subsequent activation. When UFH binds to AT-III, it produces a heparin-AT-III complex that is capable of inhibiting coagulation factors, preferably thrombin and FXa [34]. In between thrombin and FXa, thrombin has a tenfold greater sensitivity to inhibition than FXa [35]. UFH, thrombin, and AT-III must form a ternary complex for thrombin inhibition to work. However, FXa inhibition only requires UFH and AT-III to create a binary complex.

Low-molecular-weight heparin (LMWH), a subclass of heparin (MW = 3–6.5 kDa), is prepared from the fractionation or chemical depolymerisation of UFH [37]. Consequently, LMWH contains a smaller saccharide chain (typically < 18 units), making it predominantly bind to AT-III. This gives the LMWH-AT-III complex greater specificity to FXa inhibition than thrombin inhibition [35].

Vitamin K antagonists (VKAs), such as warfarin, block vitamin K epoxide reductase, an enzyme that is essential for restoring vitamin K epoxide to its active reduced form [38]. This blocking interferes with the activation of coagulation factors, such as VII, IX, X, prothrombin, and anti-clotting proteins C and S, effectively producing an anticoagulation effect [38,39].

Recently, a new class of anticoagulation drugs called direct oral anticoagulants (DOACs) have been introduced to replace traditional anticoagulants. These DOACs have several advantages over warfarin. These advantages include a broad therapeutic window, predictable pharmacological effects, minimal interactions with other medications, and no need for regular laboratory monitoring [40,41]. DOACs are classified into two categories based on their target coagulation factors (Figure 2). The first category encompasses direct thrombin inhibitors. These inhibitors function by hindering the activity of thrombin, thereby preventing the conversion of fibrinogen into fibrin. The second category comprises FXa inhibitors. These agents specifically inhibit FXa, effectively controlling the conversion of prothrombin into thrombin. Consequently, the intake of DOACs plays a pivotal role in the regulation of clot formation, maintaining balance within the coagulation cascade.

## 4. Laboratory Testing of Coagulation

The primary aim of conducting laboratory assays in haemostasis is to promptly and precisely identify dysfunctions in the complex coagulation cascade. As a result, a standardised sequence of tests is typically employed for most patients presenting coagulopathies and those under hospital care. Thus, assays involving various coagulation factors are essential for surveilling coagulation processes. These tests play a pivotal role in detecting bleeding disorders and assessing the efficacy of anticoagulant therapies.

The activated partial thromboplastin time (aPTT or PTT), prothrombin time (PT), and activated clotting time (ACT) are some of the clot-based standard techniques for the anticoagulation monitoring of patients. The aPTT assay measures how long the blood takes to form a clot. When negatively charged surfaces like kaolin, celite, silica, or ellagic acid meet FXII, they induce changes in the protein shape and activate the downstream coagulation cascade [42]. This aPTT assay measures the coagulation factors of the intrinsic pathway and proteins of the common pathway (Figure 1). The PT is another assay that measures the total time taken to form a clot when the coagulation cascade is initiated through the exogenous addition of TF. Unlike aPTT, this assay measures clotting factors from the extrinsic pathway (FVII) and common pathway proteins such as FX, FV, FII, and fibrinogen (Figure 1). The addition of exogenous TF changes the physiological concentration of coagulation factors in plasma, further activating the TF:FVIIa complex, followed by the conversion from FX to FXa [42,43]. When PT results are obtained from different laboratories using various reagents and laboratory techniques, they misrepresent PT values for warfarin therapy. To solve this issue, PT results are reported as an international normalised ratio (INR). This INR is calculated using Equation (1) [44].

INR = (PT_patient_/PT_normal_)^ISI^ (1)

where PT_patient_ is the patient sample clot time, and PT_normal_ is the geometric mean of the PT results obtained from the average population using the standard assay method, reagents, and a particular lot of thromboplastins. The supplier provides the international sensitivity index (ISI) for specific reagents. The ISI value measures each thromboplastin reagent’s response against vitamin K-dependent coagulation factors such as FII, FVII, and FX [42].

The ACT test is a whole blood-based assay, generally conducted in a near-patient or point-of-care (POC) setting. The ACT is valuable in elevated heparin concentrations, such as cardiac catheterisation and extracorporeal membrane oxygenation [43]. In such scenarios, the aPTT test cannot be used. Furthermore, due to its integration into various POC devices, the potential for a quicker turnaround time with the ACT renders it advantageous in operating rooms and cardiac care units. The ACT entails introducing a clotting activator, such as celite, kaolin, or glass beads, to a whole blood sample and then measuring the clotting time [43].

## 5. Electrochemical Sensors for Anticoagulants Based on Coagulation Factors

Traditional analytical techniques, such as mass spectrometry, provide clinicians with critical information for anticoagulant testing. Nonetheless, there exist scenarios where hospitals encounter difficulties in swiftly reporting anticoagulant drug concentrations during emergencies, which is attributable to infrastructural constraints, posing a potential threat to patient safety. An alternative strategy involving POC testing using whole blood samples has been devised in response to this challenge. This approach is designed to support clinicians in managing anticoagulation therapy more effectively. Furthermore, this POC device proves to be beneficial during emergency procedures, where immediate dosage adjustments are required, and in the ongoing, out-of-hospital monitoring of VKAs, such as warfarin, thereby enhancing patients’ clinical outcomes [45].

Most anticoagulation drug testing depends on the formation of thrombin, which converts fibrinogen to fibrin, leading to the fibrin clot. Naturally, measuring thrombin activity or directly quantifying thrombin can be used to quantify the anticoagulants that are present in the sample. From the electrochemical detection perspective, thrombin quantification can be broadly classified into two categories:

(1) When a thrombin-specific peptide containing an electroactive group is added to the sample, the hydrolysis of the peptide by thrombin releases an electroactive group, which can then be quantified by applying a respective redox potential.
(2) Thrombin can be directly quantified with the help of electrode modification by thrombin-specific aptamers; a change in the current response is a direct indication of the quantity of thrombin in the sample.

### 5.1. Electrochemical Sensors for Anticoagulants Using Thrombin-Specific Peptides

Coagucheck XS (manufactured by Roche, Basel, Switzerland) is a POC platform that is readily available for clot-based assays utilising an electrochemical detection method. Blood obtained from a finger puncture is placed onto strips containing a thrombin-specific electrogenic substrate, which releases the redox-active molecule when thrombin encounters the substrate, which is connected to a portable device that analyses the PT/INR values. Several studies utilised a Coagucheck XS coagulation analyser for the INR measurement of warfarin and LMWH [46,47,48,49]. Similarly, i-STAT (Abbott Diagnostics, Macquarie Park, Australia) and the Xprecia Stride coagulation analyser (Siemens Healthcare Diagnostics, Macquarie Park, Australia) also use a similar electrochemical platform [50,51,52,53].

A biosensor system was developed to measure the thrombin generation in a low-volume sample of as little as 10 µL of plasma or whole blood [54]. This amperometric sensor strip contains a palladium working electrode and Ag/AgCl as both a reference and counter electrode, arranged in a co-planar configuration. The test chamber was prepared with a reagent comprising a blood coagulation activator and a thrombin-specific electrogenic substrate, both of which were dried within the chamber. Following the introduction of a blood analyte, thrombin generated in situ cleaves the substrate, consequentially liberating a redox-active group. The quantity of this group that is released is directly proportional to the thrombin concentration. A comparative analysis of the results obtained using diverse activators reveals that this assay system can discern abnormalities in the extrinsic, intrinsic, and common pathways of blood coagulation.

The Coagucheck XS pro system was used to analyse the DOAC concentrations of the patients’ samples, and it was found that rivaroxaban showed an almost linear relationship with the PT values [55]. However, this method proved to be inaccurate in precisely detecting apixaban and dabigatran. It is important to note that tests designed explicitly for traditional coagulation assays, like PT/INR and aPTT, are unsuitable for the sensitive quantification of DOACs.

**Figure 3:**
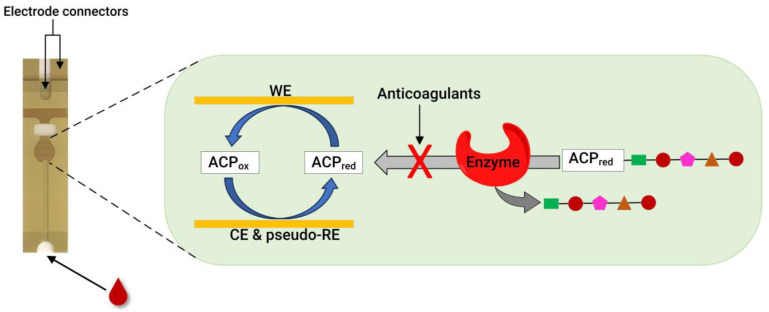
A photo of a disposable strip with gold co-facing electrodes (**left**) and a schematic diagram of co-facing gold disposable electrodes used for DOAC quantification (**right**). For the thrombin inhibitor assay, thrombin is used as an enzyme, and the substrate is Tos-Gly-Pro-Arg-ACP. Meanwhile, for the FXa inhibitor assay, FXa is used to measure the anticoagulant concentration using the Cbz-D-Arg-Gly-Arg-ACP substrate. WE = working electrode, CE = counter electrode, and pseudo-RE = Au pseudo-reference electrode. Adapted from references [56,57,58].

Recently, our group developed an assay for dabigatran using disposable gold co-facing electrodes (Figure 3), which allowed for the calibration-free quantification of the electroactive mediator concentration [56,57]. The working principle of the sensor involves measuring the activity of thrombin using the specific substrate Tos-Gly-Pro-Arg-ACP, which contains the redox active group 4-amino-2-chlorophenol (ACP). Upon the addition of thrombin, the enzyme selectively cleaves the peptide bond between arginine and ACP, thereby releasing ACP. Subsequently, the concentration of ACP is quantified by applying a specific voltage to facilitate its electrochemical oxidation. The resulting amperometric current generated during this process correlates with thrombin activity. This activity is inversely related to the presence of dabigatran. This thrombin activity-based sensor can detect ≥ 9.6 ng mL^−1^ dabigatran and quantify 11.5–140 ng mL^−1^ dabigatran when diluted plasma samples are spiked with the drug. The sensor encompasses both the therapeutic range and clinically relevant concentrations, which is significant in emergencies. Concentrations of dabigatran ≤ 30–50 ng mL^−1^ are considered safe for emergency surgeries in individuals undergoing dabigatran therapy, underscoring the importance of this sensor [56].

Much research has been dedicated to detecting thrombin as a vital coagulation factor. The aptamer-based detection of thrombin is one such area, and studies on a wide range of sensing mechanisms, including electrochemical systems, are available in the literature [59,60,61]. Therefore, discussing all studies in the literature on electrochemical aptamer-based thrombin sensors in this review is impossible. As a result, only a few examples where sensors have a linear range of detection in the order of nanomolar concentration of thrombin are discussed. This concentration range is significant because, during a coagulation phase, the quantity of thrombin can vary between 1 nM and greater than 500 nM [62,63].

A label-free method for the detection of thrombin in blood serum utilising an aptamer-based, highly specific, selective, and reusable electronic sensor has been developed [64]. The detection method employed in this study is based on a conformational change in the aptamer, which occurs selectively upon binding with thrombin. In the absence of the target molecule, the aptamer, tagged with methylene blue and covalently attached to a gold electrode, exhibits high flexibility. This flexibility facilitates a significant degree of electron transfer. Conversely, introducing thrombin leads to the formation of a rigid complex between the aptamer and thrombin. This rigidity hinders the electron transfer between methylene blue and the electrode, altering the electrochemical signal. This change in electron transfer dynamics serves as the basis for detecting thrombin. The sensor described above successfully quantified the thrombin concentration in a blood sample in the 6.4 to 768 nM range. Similarly, a thrombin detection electrochemical sensor has been created by employing a gold stripping voltammetry assay and a DNA aptamer immobilised on the nanostructured surface of the screen-printed and gold nanoparticle electrodes [65]. This sensor measured a linear range of 10 nM to 10 µM of thrombin in a phosphate-buffered saline system.

A magnetic force-assisted aptamer-antibody sandwich assay was developed based on an electrochemical detection platform for thrombin detection in serum samples [66]. In this sensor, the washing step was replaced by a magnetic fled to remove the unbound conjugate in the sample. The thrombin detection was achieved with the help of an aptamer probe, and the respective electrochemical signal was generated by the cathodic currents of toluidine blue, which were attached to the thrombin antibody-modified magnetic particle. As mentioned, the sensor detected thrombin in the serum sample and quantified it in the 1 to 500 nM range in 7 min. On the other hand, a reagentless, label-free, aptamer-based sensor for thrombin quantification was developed using a capacitive transducer with a face-to-face electrode configuration [67]. The binding of thrombin to a specific aptamer led to a sensitive change in the capacitance values used as a primary sensing mechanism. This sensor showed a linear detection range for thrombin in serum samples between 0.01 and 1000 nM. Zhu and co-workers took advantage of multivalent aptamers and developed an electrochemical thrombin sensor, which provides increased binding affinity and selectivity in target identification [68]. A fourfold increase in binding response was achieved for multivalent aptamers compared to aptamers consisting of a single recognition element. This sensor showed a dynamic range of 50 nM to 1.0 µM of thrombin in 50% diluted serum samples. To detect low concentrations of thrombin in serum, an electrochemical sensor utilising hybrid transducers comprising polypyrrole and palladium nanoparticles, with liposomes containing a redox marker employed as a label, was developed [69]. This sensor uses aptamer for thrombin recognition in samples; a linear detection range between 0.1 nM and 1 µM was obtained in the serum samples.

Similarly to the case of thrombin, monitoring FXa activity is another viable method for quantifying anticoagulants. This was confirmed by our group; by using a disposable gold co-facing recycling electrode system, an FXa enzyme-based POC assay was demonstrated for DOACs involving rivaroxaban, edoxaban, and LMWHs, such as enoxaparin and dalteparin [58]. The working principle of this sensor is similar to that described in Figure 3. The enzymatic reaction releases the ACP in the presence of the FXa-specific substrate Cbz-D-Arg-Gly-Arg-ACP peptide. However, when FXa inhibitors are present, they readily inactivate the FXa enzyme, leading to a proportional decrease in the measured amperometric current. Therefore, the measured amperometric current is related to the activity of FXa, which, in a reciprocal relationship, is influenced by the presence of the FXa inhibitor within the solution. This sensor can detect ≥ 9.00 ng mL^−1^ of rivaroxaban and quantify it within the 11.0–140 ng mL^−1^ range. In addition, the lower detection limit for edoxaban is 12.9 ng mL^−1^, and the quantification range is in the 16.8–140 ng mL^−1^ range. Likewise, enoxaparin and dalteparin (two LMWHs) were also quantified using the same sensor. A concentration ≥ 0.016 IU mL^−1^ of enoxaparin was detected, showing a quantification range of 0.025–0.75 IU mL^−1^. Meanwhile, the dalteparin detection limit was 0.013 IU mL^−1^, and the quantification range was 0.019–0.75 IU mL^−1^. With a rapid assay completion time of less than 30 s, requiring only a minimum sample volume of 7 μL, and the ability to operate at a physiological pH without calibration, this sensor is a potentially helpful candidate for POC testing.

### 5.2. Electrochemical Sensors for Anticoagulants Using Modified Electrode Surfaces

Electrode surfaces can be modified to enable an electrochemical function that is either unfeasible or challenging to achieve with standard electrodes. Specific enhancements tend to improve the electrode surfaces’ sensitivity, selectivity, and electrochemical stability. Utilising electrochemical methods to detect analytes without relying on enzymes offers numerous benefits, such as higher temperature and pH stability, simplicity, reproducibility, and cost-effectiveness [70]. Considerable progress has been achieved in synthetic methodologies, enabling the preparation of diverse materials with precise control over their size, shape, surface charge, and physicochemical attributes for electrochemical sensor applications [71,72]. Table 1 presents a summary of electrochemical sensors designed for detecting and quantifying anticoagulants using modified electrode surfaces.

An electrochemical sensor was designed to detect warfarin based on the formation of a molecularly imprinted polymer film within the multi-walled carbon nanotubes and gold nanoparticle double layers [73]. This sensor exploits the high ratio of imprinted sites and the large surface-to-volume ratio associated with carbon nanotubes and gold nanoparticles to achieve higher binding kinetics of the imprinted layer. The modified sensor showed a linear quantification range of 0.101–2.00 nM for warfarin. Similarly, a chip-based sensor containing a gold–silver alloy microwire with a 3D nanoporous surface and the molecularly imprinted polymer was used for warfarin quantification [74]. This system employed two methods for warfarin quantification based on the electrocatalysis of warfarin and the molecularly imprinted polymer gate effect. The electrochemical catalysis method exhibited linearity within the 5–400 µM range, falling short of the clinical testing requirements.

**Figure 4:**
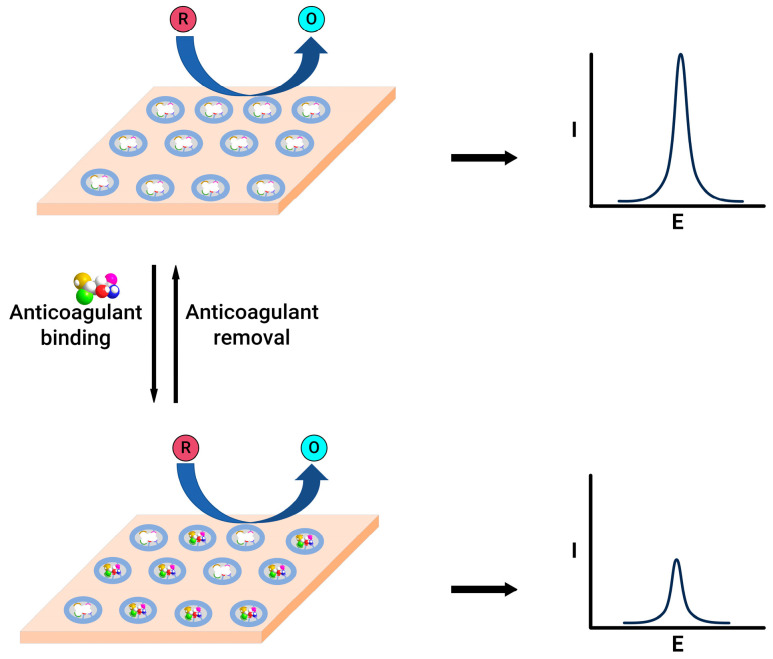
Schematic representation of molecularly imprinted polymer-modified electrodes before (**top**) and after (**bottom**) anticoagulant binding.

In contrast, the gate effect, where the alteration in the redox peak current of the redox species, observed before and after the binding of the warfarin to the electrode (Figure 4), demonstrated linearity in the 0.02–4 nM range, featuring an exceptionally low detection limit of 8 pM, effectively meeting the demands of clinical assays. A nanoporous gold leaf and molecularly imprinted polymer-modified gold electrode were used to enable the highly sensitive and specific determination of warfarin [75]. This sensor utilises a probe ion system, such as [Fe(CN)_6_]^3−/4−^. Upon binding, the warfarin into the pores of molecularly imprinted polymer leads to decreased access for electron transfer, which reduces the current. This change in the current magnitude was employed to quantify warfarin in the 0.04–80 nM range.

A glassy carbon electrode modified by cadmium sulphide quantum dots linked to chitosan and multi-walled carbon nanotubes was designed and used for warfarin quantification [76]. This sensor detected warfarin within the range of 0.05–80 μM, with a lower detection limit of 8.5 nM of warfarin, utilising a differential pulse-stripping voltammetry technique. The increased electrochemical response for warfarin compared to unmodified glassy carbon electrodes was attributed to the increased active surface area after the modification. Similarly, a nickel-doped ceria nanosphere-modified glassy carbon electrode was employed to quantify warfarin [77]. The presence of nickel-doped ceria nanospheres showed excellent electrocatalytic capability for warfarin detection with a linear range of 10 nM to 151 µM and a detection limit of 6.3 nM.

On the other hand, a carbon paste electrode sensor was also developed for warfarin detection [78]. This sensor works on the principle of the direct oxidation of warfarin and uses differential pulse voltammetry to quantify the drug. The warfarin linear quantification range was between 1.05 µM and 3 mM, showing a 0.315 µM detection limit. Similarly, a flow injection technique was developed for quantifying warfarin in pharmaceutical formulations [79]. The method relies on the drug’s susceptibility to oxidation at the glassy carbon electrode. Applying an electrode potential of +1.5 V vs. Ag/AgCl achieved a linear calibration curve in the 3.24–130 µM concentration range, with a minimum detectability of 16.2 nM.

An ion-selective electrode-based detection technique was developed to quantify warfarin in blood samples [80]. The sensing mechanism is elucidated through electrostatic interaction and coordination between warfarin and tetradodecylammonium chloride. This interaction arises from the acid–base reaction involving the acidic enol of warfarin and the ammonium group of tetradodecylammonium chloride at pH 7.4. The sensor showed good sensitivity with a detection limit of 0.125 and 14 µM of warfarin in the buffer and blood.

Heparin, an essential anticoagulant, is extensively used in clinical settings to treat thromboembolic conditions. However, patient responses to standardised doses of heparin can vary significantly, potentially leading to severe bleeding complications [95,96]. This led to the development of sensors whose primary goal is to quantify heparin and help achieve a higher degree of clinical outcomes. In the literature, some reviews discuss heparin sensors based on electrochemical detection methods [97,98,99]. However, it is necessary to integrate the recent advancements and findings in this area to provide a more current and comprehensive understanding. For example, a glassy carbon-modified electrode with a poly(thionine) thin film was developed [81]. The modified electrode displayed distinct redox peaks. Upon introducing heparin, these peaks diminished proportionally without altering the peak potential. A sensor was constructed to utilise the interaction between heparin and poly(thionine). This sensor demonstrated a robust response within the 0.27–1.47 µM concentration range, achieving a detection limit of 18.7 nM. Additionally, polyethyleneimine was used as a receptor, and surface-confined ferricyanide was used as a redox probe to construct a heparin sensor [82]. The strong attraction of polyethyleneimine to heparin facilitates the anionic exchange between the confined ferricyanide and the larger pool of heparin in the test solution. This sensor showed a linear range of 0.133–2.67 µM in 0.1 M of phosphate-buffered saline. However, this work did not use human plasma-spiked samples to show the applicability of this sensor for clinical settings.

A simple voltammetric platform was developed for heparin detection [100]. The sensor system consisted of gold nanoparticles and a γ-substituted pentamethinium salt-modified glassy carbon electrode covered by a plasticised polyvinyl chloride-based membrane. The selectivity was achieved by interacting with positively charged γ-substituted pentamethinium and negatively charged heparin. The suggested voltammetric sensor demonstrated a concentration correlation ranging from 2.23 to 60.0 µM of heparin. It was applied to detect heparin in saline buffer and biological samples, achieving a recovery rate between 95.1% and 100.9%.

Similarly, the interaction between protamine, a heparin antidote, and heparin was evaluated using a gold-decorated graphene oxide glassy carbon electrode system [83]. This sensor utilises a probe ion system such as [Fe(CN)_6_]^3−/4−^; upon the binding of heparin to protamine, the decrease in the peak current was correlated to an increase in the heparin concentration. The linear range of heparin quantification was between 1.59 nM and 19 µM with a 0.9 nM low detection limit. A molecularly imprinted polymer grafted onto the graphite paste electrode surface was also presented [101]. The incorporated particles were extensively blended with oil to produce the molecularly imprinted polymer–graphite paste electrode. Standard cyclic voltammetry was conducted using the electrode in saline buffer or bovine whole blood, incorporating 5 mM of ferrocyanide, which showed a linear quantification of heparin ranging from 0 to 10.7 µM. The current intensity rose in tandem with the concentration of heparin, which was attributed to the enlargement in the effective surface area caused by the increased mobility of the oil in the molecularly imprinted polymer–graphite electrode facilitated by heparin.

An aptamer-based sensor for detecting dabigatran etexilate was also developed [84]. An aptamer with high affinity and specificity was chosen as a sensing probe in addition to the reporting probe [Fe(CN)_6_]^3−/4−^ redox system. The square wave voltammograms showed a good relationship between the dabigatran etexilate concentrations and the decreased peak current of the [Fe(CN)_6_]^3−/4−^ redox molecule. The reduction in the peak current after the dabigatran binding could be due to the more compact arrangement between the aptamer and drug. This hindered the access of the redox molecule to the electrode surface. Even though this work was not carried out in the plasma or blood samples, this sensor showed a good response in the 15.9 pM to 1.59 µM range in the phosphate-buffered saline. On the other hand, an electrocatalytic sensor consisting of graphene and cerium dioxide-modified glassy carbon electrode was prepared to identify and quantify dabigatran etexilate [85]. The square wave voltammograms showed that when the dabigatran concentration increased, the cathodic current also increased linearly within the 7.97 nM–7.97 µM range; this was attributed to the good electrocatalytic activity enhancement of the modified electrode.

An electrochemical sensor for dabigatran etexilate in the pharmaceutical and urine samples was designed using a boron-doped diamond electrode (BDDE) and a basal-plane pyrolytic graphite electrode (BPPGE) [86]. This sensor exploited the irreversible oxidation of dabigatran etexilate and obtained a concentration-dependent calibration curve with respect to the oxidation current in a pH 3.0 buffer system. The sensor showed linear detection ranges between 0.01 and 0.7 µM of dabigatran using BDDE and between 0.07 and 1.0 µM of dabigatran with BPPGE. To test its versatility, an analysis of the spiked urine and pharmaceutical samples was employed using the standard addition method, which resulted in a good response without any significant interference. In comparison to BPPGE, the BDDE system performed better. Similarly, an ion-selective sensor was developed based on modified carbon paste and pencil graphite electrodes consisting of a dabigatran etexilate–phosphotungstate ion pair [87]. These modified electrodes quantified dabigatran etexilate in the 10 µM–10 mM range for the ion-selective carbon paste electrode and in the 1 µM–10 mM range when the pencil graphite electrode was used.

A new label-free electrochemical aptasensor was created by linking a thiolated aptamer with gold nanoparticles onto the surface of indium tin oxide-polyethylene terephthalate (ITO-PET) [88]. This aptasensor was effectively utilised to quantify rivaroxaban in human plasma and exhaled breath condensate samples, achieving detection limits of 14.08 and 6.03 nM, respectively. The square wave voltammetry method assessed the sensor’s analytical performance in a [Fe(CN)_6_]^3−/4−^ redox solution. The electrochemical signal gradually rose with an increasing rivaroxaban concentration, establishing a robust linear relationship between the current response signal and rivaroxaban concentration. The calibration curve demonstrated linearity within the 10–600 nM concentration range in plasma and exhaled breath condensate samples. A similar approach was designed using ITO-PET modified by polytoluidine blue, silver nanoparticles, and polyethylene glycol as electrode substrates and aptamers as rivaroxaban-recognising elements [89]. This sensor showed 6.65 and 4.13 nM detection limits for the plasma and exhaled breath condensate samples.

On the other hand, a potentiometric sensor was developed by modifying a glassy carbon electrode [90]. The sensor required the integration of a molecularly imprinted polymer into polyvinyl chloride with a plasticiser and its application as a singular layer on the glassy carbon electrode. Similarly, a voltammetric sensor was crafted through a drop-coating method, sequentially depositing graphene oxide and a molecularly imprinted polymer on the untreated glassy carbon electrode. The rivaroxaban potentiometric sensor showed a linear response range between 1.2 nM and 1.0 mM. However, a linear range of 0.054 nM–3.1 mM was obtained using the voltammetric sensor. Both sensors showed good responses for rivaroxaban in the blood and pharmaceutical samples.

An electrochemical sensor for rivaroxaban in the pharmaceutical and urine samples was designed using a boron-doped diamond electrode (BDDE) and graphite flake paste electrode (GFPE) [86]. This sensor operates based on the irreversible oxidation of rivaroxaban. As a result, a calibration plot of the increases in the anodic current and rivaroxaban concentration was plotted. The calibration ranges for rivaroxaban were 0.5–30.0 µM for BDDE and 1.0–10.0 µM for GFPE. To test the sensor’s performance, an analysis of the spiked urine and pharmaceutical samples was employed using the standard addition method, which resulted in a good response without any significant interference.

A potentiometric sensor consisting of a glassy carbon electrode coated with an ion-selective membrane and multi-walled carbon nanotubes was developed to measure edoxaban [91]. This modification improved the sensor’s performance, including an expanded dynamic range from 6.0 µM to 1.0 mM of edoxaban and a reduced detection limit of 4.20 µM of edoxaban. Moreover, this sensor was also evaluated using a pharmaceutical formulation and edoxaban-spiked human plasma samples. Similarly, a sensor chip based on a carbon paste electrode embedded with molecularly imprinted polymers and p-styrene sulfonate was developed to detect and quantify edoxaban [102]. The interaction of edoxaban through the cavities created inside the grafted layer enhances the oxidation current of ferrocene, which is used as the redox probe. Additionally, p-styrene sulfonate has a greater affinity for edoxaban, improving the sensor’s current response. Nevertheless, the poor sensitivity and selectivity reported prevent the sensor’s practical application in clinical settings.

The surface of the glassy carbon electrode was modified with graphene oxide aerogels and a Bi_2_Fe_4_O_9_ semiconductor to design an apixaban sensor [103]. This sensor used a [Fe(CN)_6_]^3−/4−^ redox system as the reporting probe. Upon the addition of apixaban to the plasma sample, the redox current of [Fe(CN)_6_]^3−/4−^ decreased due to apixaban blocking the electrode surface. This correlation between the decreasing peak current and the increasing apixaban concentration showed a linear range of 10 ng mL^−1^ to 10 µg mL^−1^. Additionally, a carbon paste electrode modified with multi-walled carbon nanotubes was prepared for the voltammetric determination of apixaban in pharmaceutical compounds [94]. At pH 5.0, apixaban showed an oxidation process at 1.21 V vs. the Ag/AgCl reference system. The peak current vs. the concentration of apixaban resulted in a linear detection range between 1.99 and 107 µM of apixaban. The apixaban-spiked plasma test returned a 100.64 ± 1.22% recovery rate.

## 6. Common Calibration Methods

Most commercially available FXa inhibitor assays depend on measuring the FXa activity upon the in vitro addition of an inhibitor-containing sample. Present methodologies for evaluating FXa inhibitors demand distinct calibrations tailored to each specific drug, making it impossible to concurrently measure all types of FXa inhibitors, including DOACs and LMWHs [104]. This poses a notable challenge, especially for individuals undergoing FXa inhibitor treatment who are admitted to the emergency department due to severe bleeding. It is crucial to ascertain their anticoagulation statuses before considering any surgical interventions. The challenge intensifies when the patient’s consciousness is impaired, leaving their anticoagulation state indeterminate. In response to this dilemma, our recent investigations have considered the feasibility of substituting the drug-specific calibration requirements with a unified calibration strategy, encompassing a singular calibration curve for DOACs and another for LMWHs. This approach aims to enable the quantification of inhibitors within both the DOAC (e.g., rivaroxaban and edoxaban) and LMWH (e.g., enoxaparin and dalteparin) categories [58]. Our findings indicate that this sensor can accurately identify and measure the levels of rivaroxaban and edoxaban without the need for individual calibration curves or a universal calibration scheme, achieving an acceptable margin of error, even when the specific identity of the inhibitor is unknown, with results displaying an error margin of ±3 ng/mL. Extending this concept to LMWHs has also shown promising results; a statistical analysis revealed no significant differences in the measurements between two distinct LMWHs, suggesting the potential for a universal calibration curve to accurately assess both types of inhibitors with minimal error. Nonetheless, this innovative approach requires further validation across a broader spectrum of DOACs and LMWHs with diverse molecular sizes to confirm its efficacy and applicability in POC contexts.

## 7. Conclusions

This review article provides a comprehensive synthesis of the advancements in the electrochemical monitoring of anticoagulation therapy, critically analysing the current state of the art against the backdrop of previous research findings. By aggregating and examining diverse studies, we highlight a significant progression in sensor sensitivity, specificity, and the pragmatic utility of universal calibration methods for DOACs and LMWHs. These enhancements are not merely incremental; they represent substantial strides from traditional monitoring methodologies, addressing longstanding challenges in anticoagulation management.

The critical analysis within this review underscores the paramount importance of precise anticoagulant monitoring, which is pivotal in navigating the narrow therapeutic windows inherent to anticoagulant use. The improved detection limits and quantification accuracy, as discussed above, have clear clinical implications. They offer the potential for more refined dosing strategies, reducing the risk of under- and over-anticoagulation and thereby minimising adverse events.

Moreover, as explored in the literature, the introduction and validation of universal calibration plots suggest a promising direction for simplifying and accelerating the monitoring process. This advancement could greatly enhance the practicality of POC testing, making it more accessible and efficient. Such improvements are critical in emergency settings, where the rapid determination of the anticoagulation status can be lifesaving.

By situating our findings within the broader research landscape, we advocate for the integration of these advanced electrochemical monitoring technologies into routine clinical practice. This review not only catalogues technological advancements but also calls for a paradigm shift in how anticoagulant therapy is monitored, emphasising the need for a balance between innovation and patient safety.

In synthesis, this article aims to serve as a cornerstone for future investigations, guiding researchers and clinicians towards optimising anticoagulation therapy. The ultimate goal is to enhance patient care by adopting these cutting-edge monitoring techniques, which promise to reduce complications and improve patient outcomes in anticoagulant therapy.

## Figures and Tables

**Table 1:** Electrochemical sensors designed for detecting and quantifying anticoagulants using modified electrode surfaces.

Sensor Composition	Anticoagulant Detected	Detection Limit	Quantification Limit	Ref.
AuNP/MIP/f-MWCNT/GCE	warfarin	0.078 nM	0.101 nM	[73]
MIP/NPAMW	warfarin	8.00 pM	20 pM	[74]
MIP/NPGL/GE	warfarin	41.0 pM	NA	[75]
CdS-QDs/CS/MWCNTs/GCE	warfarin	8.50 nM	28 nM	[76]
CeO_2_@Ni/GCE	warfarin	6.30 nM	10 nM	[77]
CPE	warfarin	0.315 µM	1.05 µM	[78]
GCE	warfarin	16.0 nM	3.24 µM	[79]
TDDA/ISE	warfarin	14 µM (blood)	NA	[80]
		0.125 µM (buffer)	NA	
poly(thionine)/GCE	heparin	18.7 nM	0.27 µM	[81]
[Fe(CN)_6_]^3−^/PEI/SWCNT-GCE	heparin	0.088 µM	0.133 µM	[82]
GO/Au-protamine/GCE	heparin	0.9 nM	1.59 nM	[83]
Aptamer/GE	dabigatran	15.9 pM	NA	[84]
GR/CeO_2_/GCE	dabigatran	19.9 nM	66.3 nM	[85]
BDDE	dabigatran	2.78 nM	9.25 nM	[86]
BPPGE		16.7 nM	55.8 nM	[86]
ISCPEs	dabigatran	4.36 µM	NA	[87]
ISPGEs		0.426 µM		
Aptamer-AuNPs-PET-OH-ITO	rivaroxaban	14.1 nM (plasma) 6.03 nM (EBC)	NA	[88]
ITO-PET-OH-p(TB-Ag)-Aptamer-PEG	rivaroxaban	6.65 nM (plasma) 4.13 nM (EBC)	NA	[89]
MIP/PVC/GCE	rivaroxaban	2.29 µM	6.88 µM	[90]
GCE/MWCNT-ISM	edoxaban	3.39 µM	NA	[91]
PGE	edoxaban	0.073 µM	0.243 µM	[92]
GCE	edoxaban	0.24 µM	0.84 µM	[93]
BDDE		0.57 µM	1.90 µM	
MWCNT/CPE	apixaban	0.618 µM	1.99 µM	[94]

AuNP: gold nanoparticles; MIP: molecularly imprinted polymer; f-MWCNT: carboxyl functional group containing multi-walled carbon nanotubes; GCE: glassy carbon electrode; NPAMW: 3D nanoporous gold–silver alloy microwire; NPGL: nanoporous gold leaf; GE: gold electrode; CdS-QDs: cadmium sulphide quantum dots; CS: chitosan; MWCNTs: multi-walled carbon nanotubes; CPE: carbon paste electrode; TDDA: tetradodecylammonium chloride; ISE: ion-selective electrode; PEI: polyethyleneimine; SWCNT: single-walled carbon nanotube; GR: graphene; BDDE: boron-doped diamond electrode; BPPGE: basal plane pyrolytic graphite electrode; ISCPEs: ion-selective carbon paste electrodes; ISPGEs: ion-selective pencil graphite electrodes; PET-OH: hydroxylated polyethylene terephthalate; ITO: indium tin oxide; EBC: exhaled breath condensate; PTB: poly toluidine blue; PEG: polyethylene glycol; PVC: polyvinyl chloride; ISM: ion-sensing membrane; PGE-pencil graphite electrode.

## Data Availability

Not applicable.
